# Cytotoxicity and Immune Dysfunction of Dendritic Cells Caused by Graphene Oxide

**DOI:** 10.3389/fphar.2020.01206

**Published:** 2020-08-17

**Authors:** Zhiwen Yang, Yongning Pan, Tingting Chen, Li Li, Wenyi Zou, Dongmeng Liu, Dahui Xue, Xiaomei Wang, Guimiao Lin

**Affiliations:** ^1^ Base for International Science and Technology Cooperation: Carson Cancer Stem Cell Vaccines R&D Center, Shenzhen Key Lab of Synthetic Biology, Department of Physiology, School of Basic Medical Sciences, Shenzhen University, Shenzhen, China; ^2^ Department of Disease Prevention and Control, Shenzhen Baoan District Health Bureau, Shenzhen, China

**Keywords:** graphene oxide, immunotoxicity, reactive oxygen species, dendritic cells, cytokines

## Abstract

Graphene, known as “black gold”, has important applications in various fields. In previous studies, it has been proved that graphene oxide (GO) which is a derivative of graphene has low toxicity. However, the immunotoxicity of GO has not been fully elucidated. In this work, we used DC2.4 cell line to investigate the *in vitro* immunotoxicity of two types of GO, mono-layer GO (mono-GO) and multi-layer GO (multi- GO). We found that mono-GO had less effect on cell viability than multi-GO, but both mono-GO and multi-GO significantly induced the generation of ROS in DC2.4 cells. Interestingly, mono-GO caused DC2.4 cells to aggregate, thus changed the cell morphology significantly. However, no similar influence occurred for multi-GO. In addition, the results showed that these two GOs obviously enhance the release of TNF-α by DC2.4 cells with and without LPS stimulation. GO did not affect the level of IL-6 released from DC2.4 cells, but multi-GO promoted the release of IL-6 while mono-GO inhibited the production of IL-6 when cells were in response to LPS stimulation. Whole-transcriptome sequencing analysis found some immune-related differentially expressed genes including H2-DMb1, Ncbp3, Oas2, Men1, Fas, Cd320, Cd244, and Tinagl1 which are engaged in the immune system process. These results suggested that both mono-GO and multi-GO are immunotoxic to DC2.4 cells, which provides important basis for subsequent biological and clinical medical applications.

## Introduction

Graphene, called the “21st century materials”, is a 2D nanomaterial composed of a single layer of sp2 carbon atoms (Carrow et al., 2018). Graphene can be divided into different types according to its functional form (Novoselov et al., 2004). Among them, graphene oxide (GO) is an oxidized form of graphene containing various oxygen-containing functional groups, such as carboxyl, carbonyl and hydroxyl (Gao, 2015). Since GO has a large surface area, and good physical, chemical and biological properties (Reina et al., 2017), they are considered for biological applications such as bioimaging, diagnostics, biosensing, photothermal and photodynamic therapy, tissue engineering, and drug delivery (Nurunnabi et al., 2015). Han et al. demonstrated that GO can be used as natural antioxidants to reduce inflammation by reducing reactive oxygen species (ROS) in macrophages and used as a gene carrier to synergistically treat myocardial infarction (Han J. et al., 2018). In addition, Zhang et al. proposed a GO-based fluorescent nanosensor that can be used to quickly detect telomerase content in cells and image telomerase (Zhang et al., 2018). Zhou et al. studied a new method of introducing GO into bone tissue to enhance biomineralization, and they found that when the GO concentration was 0.1% w/v and immersed in simulated body fluids for 7 days, GO-collagen-apatite 3D scaffold showed good therapeutic effect in repairing skull defects in rats (Zhou et al., 2018).

Although GO has a good application prospect, its unknown toxicity is still a bottleneck to clinical application. In addition, with the widespread use of GO, there are a variety of indirect and direct pathways to introduce them into the environment (e.g., dust or wastewater) (Markovic et al., 2018). Therefore, studies on the toxicity of GO need to be urgently addressed. In the recent years, Qu et al. summarized the toxic effects of graphene-family nanomaterials (GFNs) in cell and organs models and pointed out that GO can be transmitted to bodies through oral administration, intraperitoneal injection and intravenous injection and thus cause various inflammatory reactions (Ou et al., 2016). The toxicological mechanisms of GFNs mainly contain inflammatory response, necrosis apoptosis, DNA damage, and autophagy etc. For example, grapheme oxide caused a significant increase in intracellular ROS production in human HaCaT skin keratinoyctes, which was mainly mediated by activation of flavin-based oxidase, and caused mitochondrial membrane depolarization and mitochondrial damage (Pelin et al., 2018). Although many *in vitro* and *in vivo* toxicity studies of graphene have been reported, little research has been done on the immune system.

The immune system plays an important role in identifying and eliminating foreign pathogens. Immune cells can directly contact nanomaterials and are responsible for potential adverse reactions to GO. The results of research on the interaction between nanomaterials and the immune system can better assess the impact of GO on the human body. Recently, publications on the effects of GO on the immune system have focused on the study of macrophages (Dudek et al., 2016). Luo et al. found that PEGylated GO nanosheets interacted with peritoneal macrophage surface receptors by adsorbing to and/or partially inserted into the cell membrane, and stimulated peritoneal macrophages to produce cytokine responses (Luo et al., 2017). Hoyle et al. found that GO had no significant inflammatory response to macrophages cells. However, GO inhibited Toll-like receptor 4 (TLR4) receptor-mediated interleukin production, but did not inhibit activation of inflammatory bodies (Hoyle et al., 2018). However, little is known about the effect of GO on dendritic cells (DCs), which were the most powerful professional antigen-presenting cells (APCs). Li et al. found that GOx nanosheets could be used to adsorb proteins. When the ovalbumin antigen binds to GOx, it could be internalized by DCs and initiated the antigen presentation reaction. Therefore, GOx nanosheets could be used as nanocarriers in vaccine formulations (Li et al., 2016). DCs drive specific responses through the adaptive immune system and plays a major role in innate immunity. DCs can efficiently ingest, process, and present antigens. Immature DCs have a strong ability to migrate. Mature DCs highly express MHC molecules and co-stimulatory molecules, and activate the native T cells, which in turn causes an immune regulatory response (Qian and Cao, 2018). DCs recognizes microbial components such as lipopolysaccharide (LPS) and specifically binds TLR4 on DCs (Chen et al., 2020). After LPS binds to TLR4, DCs secrete inflammatory factors such as tumor necrosis factor-α (TNF-α) and interleukin-6 (IL-6) (Fu et al., 2011; Chen et al., 2020). Accordingly, here we studied the immunotoxicity of mono-GO and multi-GO on DCs.

In this work, we utilized dendritic cell (DCs) line DC2.4 cells as an *in vitro* model to study the immunotoxicity of mono-GO and multi-GO. We found that multi-GO caused stronger toxicity to DC2.4 cells than mono-GO. And they all promoted the production of ROS in DC2.4 cells. Interestingly, mono-GO will cause a significant change in cell morphology, compared to multi-GO. In addition, mono-GO and multi-GO alone did not cause DC2.4 cells to produce IL-6, but stimulated DC2.4 cells to produce TNF-α. However, after pretreatment with mono-GO and then treating DC2.4 cells with LPS, it was found that the cytokines secreted by DC2.4 cells were disordered. Also, we utilized RNA-seq found that both mono-GO and multi-GO were able to arouse immune responses. Our results showed that GO were able to disturb the immune function of DCs, which provided a basis for further applications and research.

## Materials and Methods

### Preparation and Characterization of GO

Mono-GO (777676) and multi-GO (796034) were purchased from Sigma-Aldrich Technology. The absorption spectra of mono-GO and multi-GO were determinated by UV-Vis spectrophotometer (Beckman Coulter, DU720) and the morphology images of mono-GO and multi-GO in water were obtained with a transmission electron microscope (TEM) (JEOL model jem-2100, Test Center of Wuhan University). Zetasizer Nano ZS device (Malvern, U.K.) was used to measure the particle size and potential.

### Cell Culture

The mouse dendritic cell line (DC2.4) were obtained in our lab. The DC2.4 cell line was cultured in RPMI 1640 (Gibco, USA) supplemented with 10% fetal bovine serum (FBS, Gibco, USA) and 100 U penicillin/streptomycin (PS, Gibco, USA). All cells were cultured at 37°C in humidified atmosphere with 5% CO_2_. One μg/ml of lipopolysaccharides (LPS, L4391, Sigma, USA) in PBS was used as a positive control to stimulate the DC2.4 cells to secrete inflammatory factors.

### Cell Viability

The cell viability of DC2.4 cells treated with mono-GO and multi-GO was determined by MTT (Thiazolyl Blue Tetrazolium Bromide, M5655, Sigma, USA) assay. Briefly, DC2.4 cells were seeded in two 96−well plates at a density of 1.5× 10^4^ cells per well. Twelve hours later, 0.01, 0.1, 1, 10, or 100 μg/ml mono-GO and multi-GO in water were added to 96-well plates. After 24 h or 48 h incubation with cells, 20 μl of MTT at a concentration of 5 mg/ml in PBS was added to each well, and the DC2.4 cells were subsequently cultured for 4 h in a cell incubator. The supernatant was then gently aspirated and 150 μl dimethylsulfoxide (DMSO, D2650, Sigma, USA)was added to dissolve the pellet. The 96-well plates were placed on a shaker and shaken for 5 min at room temperature. Finally, we used a microplate reader (BioTek, USA) to measure the absorbance of each well at a wavelength of 490 nm. All experiments were repeated three times. The cell viability was calculated by the absorbance of the sample wells against the control wells, assigning the viability of non-treated cells as 100%.

### Enzyme-Linked Immunosorbent (ELISA) Assay

To analyze the cytokine secretion by DC2.4 cells, 1×10^5^ DC2.4 cells were seeded in 24-well plates-and cultured for 24 h. Two kinds of GOs were added in 1 ml RPMI 1640 with 10% FBS and added to cells in each well at different concentrations, respectively0, 0.01, 0.1, 1, 10, 100 μg/ml, then the cells were incubated for 4 h and then 1 μg/ml Lipopolysaccharide (LPS) was added. So far, the experiment was divided into six groups: negative control group, LPS positive control group, mono-GO group, multi-GO group, mono-GO+LPS group and multi-GO+LPS group. After the DC2.4 cells were co-cultured for 24 and 48 h, the supernatant from the above six groups was collected and the cytokines (TNF-α, IL-6) were measured by mouse ELISA kits (88-7324-88 and 88-7064-88, Invitrogen, Thermo Fisher Scientific) according to the manufacturer’s instructions.

### DC2.4 Cells Morphology

First, 2.5×10^5^ DC2.4 cells were seeded in a 12-well plate. After 12 h, DC2.4 cells were treated with 10 μg/ml mono-GO for 24 h, and then rinsed briefly in phosphate-buffered saline (PBS). Next, 4% paraformaldehyde was used to fix the DC2.4 cells for about 10 min and ice-cold PBS was used to wash the cells. And then, DC2.4 cells were incubated with 0.1% Triton X-100 for about 10 min and incubated with phalloidin-FITC (P5282, sigma, USA) antibody in PBS for 40 min at room temperature in the dark, and then washed with PBS three times with 5 min for each wash. Finally, DC2.4 cells were treated with DAPI for 1 min and images were obtained with a fluorescence microscope (X-Cite Series 120, Zeiss, Germany).

### Measurement of Reactive Oxygen Species (ROS) in Dendritic Cells

ROS production by DC2.4 cells exposed to mono-GO and multi-GO was evaluated by the 2, 7-dichlorofluorescindiacetate (DCFDA) (D6883, Sigma, USA) assay. Briefly, 2× 10^4^ DC2.4 cells were seeded in 96-well clear bottom black side plate and cultured at 37°C. After 12 h, 20 μM DCFDA probe in serum-free medium was added to different wells every 30 min as a control group. At the same time, DCFDA probe and 10 μg/ml mono-GO or 10 μg/ml multi-GO in serum-free medium were added to different wells every 30 min as two experimental groups. Next, a fluorescence microplate reader (Infinite 200 Pro, Tecan) was used to read fluorescence at an excitation wavelength of 488 nm and an emission wavelength of 525 nm. The average fluorescence intensity of intracellular DCF reflected the production of intracellular ROS.

### Whole-Transcriptome Sequencing Analysis (RNA-Seq)

DC2.4 cells were cultured in 6-well plate at a density of 3× 10^5^ cells per well in 2 ml of RPMI 1640 with 10% FBS. After 12 h, DC2.4 cells were treated with 10 μg/ml mono-GO and multi-GO for 24 h, respectively. The supernatant was removed and the cells were washed twice with ice-cold PBS, then the cells were lysed in 500 μl TRIzol reagent (MRC, USA) to extract RNA, according to the manufacturer’s protocol. Then Agilent 2100 Bioanalyzer (Agilent Technologies, Palo Alto, CA, USA) was used to assess RNA integrity and quantify RNA concentration. The screening conditions for RNA samples are: 28S/18S rRNA band intensity is 2:1, spectral A260/A280 nm ratio is 1.8–2.0 and A260/A230 nm ratio is greater than 1.5. The next step is to construct a gene library. In short, magnetic beads with Oligo (dT) are used to enrich mRNA. Subsequently, the mRNA is broken into short fragments, and the mRNA is used as a template for reverse transcription to synthesize the first-strand cDNA and then the second-strand cDNA. Then use AMPure XP beads to purify the double-stranded cDNA, modify the ends to connect the sequencing adapter, and finally perform PCR amplification to obtain the final gene library. Finally, the Illumina high-throughput sequencing platform (HiSeq/MiSeq) was used for paired-end sequencing. Finally, samples were sent to Guangzhou MAGIGENE Gene Corporation for subsequent analysis. Genes which p< 0.05 and log_2_ fold change < -1 or > 1 were considered to be statistically significant and were filtered out for subsequent analysis between mono-GO, multi-GO and untreated groups. Gene Ontology (GO) enrichment analysis contains three GO terms which are biological processes (BP), molecular functions (MF), and cellular components (CC). In our study, the Kyoto Encyclopedia of Genes and Genomes (KEGG) database was used to analyze which important pathways of DCs can be changed by two GOs.

### Statistical Analysis

We used SPSS 13.0 software for statistical analysis. Statistical evaluations were performed using independent t-test and one-way analysis of variance (ANOVA) test when normality and homogeneity of variance are satisfied. The results are presented as mean ± SD values. All tests were two-sided and P<0.05 was considered statistically significant.

## Results

### Characterization of GO

**Figure 1:**
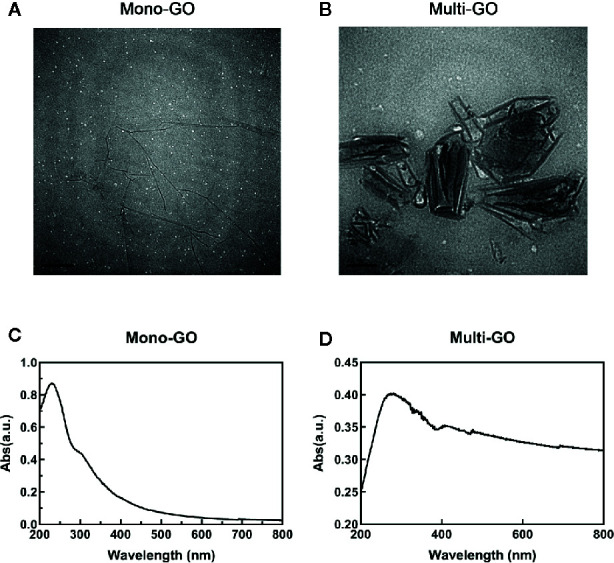
Characterization of mono–graphene oxide (GO) and multi-GO. **(A)** Transmission electron microscope (TEM) image of multi-GO. Scale bar: 100 nm; **(B)** TEM image of mono-GO. Scale bar: 200 nm; **(C)** UV-Vis spectrum of multi-GO; **(D)** UV-Vis spectrum of mono-GO.

The mono-GO and multi-GO were characterized with UV-Vis and TEM. The absorption spectrum of mono-GO was shown in **Figure 1A**. The absorption spectrum of multi-GO was shown in **Figure 1B**. As is shown in **Figures 1A, B**, the multi-GO has a wider absorption band than the mono-GO. The TEM image of mono-GO and multi-GO was shown in **Figures 1C, D**. It demonstrated that the lateral size of mono-GO is larger than that of multi-GO. It is clearly seen that the multi-GO is relatively thick due to stacking together, and the mono-GO is thin and transparent. The lateral size of mono-GO was larger than multi-GO. In order to more clearly study the differences between mono-GO and multi-GO, we used a Zetasizer Nano ZS device (Malvern, U.K.) to measure the particle size and potential. Research showed that the zeta potentials of mono-GO and multi-GO were -28.5± 0.70 and -33.4± 0.41, respectively. The zeta potential values of our graphene oxides are similar to those found by other authors (Li et al., 2014; Hidalgo et al., 2015) The results indicated that mono-GO and multi-GO were relatively stable in water. The particle sizes of mono-GO and multi-GO were 1554.00± 543.34 nm and 123.48± 47.95 nm, respectively. The results show that the particle size of mono-GO is much larger than that of multi-GO.

### Cytotoxicity Assay

**Figure 2:**
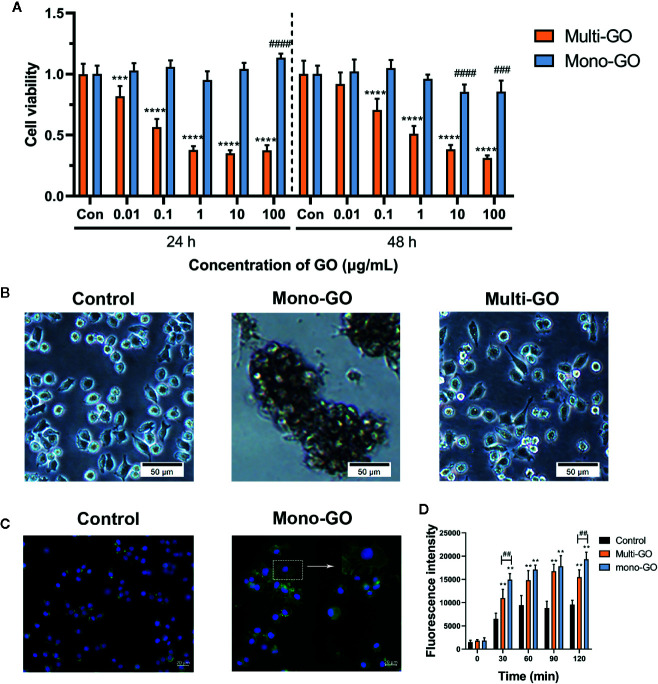
The effect of graphene oxide (GO) on the viability and morphology of DC2.4 cells. **(A)** The effect of mono-GO and multi-GO on the viability of DC2.4 cells for 24 h and 48 h. n = 10. ***P<0.001, ****P<0.0001 multi-GO vs. Control; ^###^P<0.001, ^####^P<0.0001 mono-GO vs. Control. **(B)** Representative brightfield images of DC2.4 cells treated with phosphate-buffered saline (PBS), 10 μg/ml mono-GO, and 10 μg/ml multi-GO. Scale bar = 50 μm. **(C)** Representative fluorescent picture of DC2.4 treated with control and mono-GO after phalloidin-FITC staining. Scale bar = 20 μm. **(D)** The production of reactive oxygen species (ROS) in DC2.4 cells treated with multi-GO and mono-GO within 120 min. (**P < 0.01 vs. Control; ^##^P < 0.01 vs. Multi-GO).

The effect of mono-GO and multi-GO on DC2.4 cell viability was evaluated by MTT assay after 24 and 48 h exposure time. As shown in **Figure 2A**, treatment of DC2.4 cells with different concentrations of mono-GO (0.01, 0.1, 1, 10, 100 μg/ml) for 24 h had no significant effect on the viability of DC2.4 cells. However, the viability of DC2.4 cells with mono-GO treatment for 48 h decreased obviously when the concentration reached up to 10 μg/ml. Different from mono-GO DC2.4 cell viability after multi-GO treatment decreased remarkably when the concentration was higher than 0.01 μg/ml. With the increasing concentration of multi-GO, the cell viability decreased significantly on dose-dependent manner. These results suggested that mono-GO had lower toxicity effect on DC2.4 cell viability than multi-GO.

The effect of GO on the morphology of DC2.4 cells was observed with a microscope. As shown in **Figure 2B**, after DC2.4 cells were exposed to 10 μg/ml mono-GO for 24 h, the cells tend to aggregate and the cell morphology changed obviously. However, DC2.4 cells with multi-GO treatment showed no similar cell morphology alteration (**Figure 2B**). Therefore, we further observed the cytoskeleton of mono-GO treated cells by phalloidin-FITC immunofluorescence assay. We found that the cells became larger and the cytoplasm became fuller after mono-GO treatment in comparison with those without treatment (**Figure 2C**).

To deeply investigate the effects of mono-GO and multi-GO on DCs, the kinetics of ROS production was investigated at increasing intervals of time (30 to 120 min exposure) using a time-dependent DCFDA assay. As is shown in **Figure 2D**, when compared with control, ROS production by DCs treated with mono-GO and multi-GO significantly increased at the concentration of 10 μg/ml. In addition, GO-induced ROS production was time-dependent from 30 min to 120 min. Interestingly, mono-GO-induced ROS production was more than multi-GO after 30 and 120 min exposure. These results suggested that mono-GO and multi-GO had the potential to induce the intracellular ROS generation.

### Cytokine Secretion Assay of DC2.4 Cells Exposed to Mono-GO and Multi-GO

**Figure 3:**
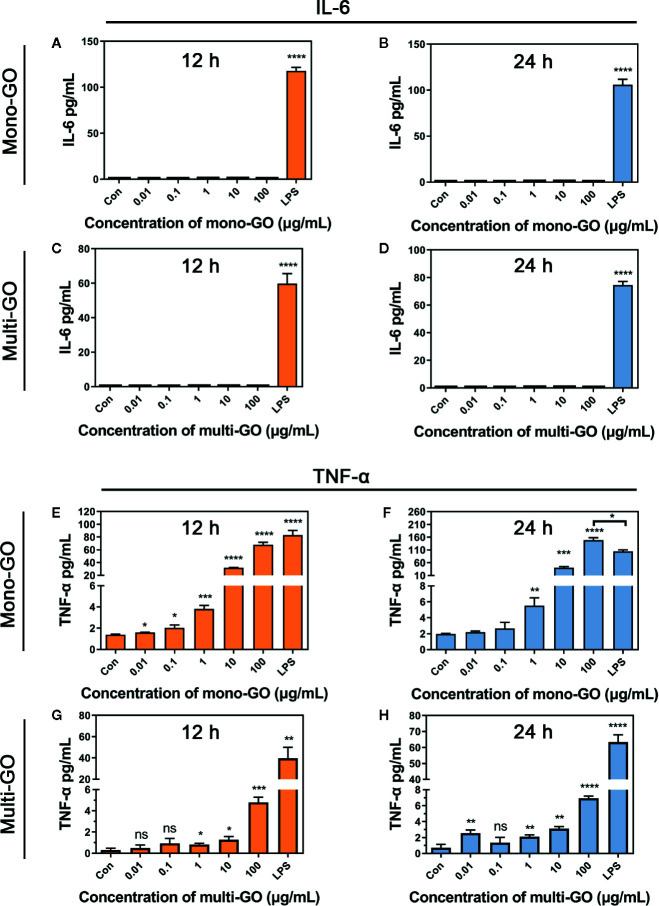
Cytokine secretion of DC2.4 cells exposed to mono–graphene oxide (GO) and multi-GO. **(A**, **B)** Cytokine IL-6 secretion from DC2.4 cells after exposure to mono-GO for 12 h and 24 h. **(C, D)** Cytokine IL-6 secretion from DC2.4 cells after exposure to multi-GO for 12 h and 24 h. **(E, F)** Cytokine TNF-α secretion from DC2.4 cells after exposure to mono-GO for 12 h and 24 h. **(G, H)** Cytokine TNF-α secretion from DC2.4 cells after exposure to multi-GO for 12 h and 24 h. (n = 3; * P < 0.05, ** P < 0.01, *** P < 0.001, **** P < 0.0001 vs. Control; ns, no significance).

To further observe the effect of mono-GO and multi-GO on the function of DCs, the production of the pro-inflammatory cytokine IL-6 and TNF-α were tested in the culture supernatants of DC2.4 cells after 24 h-exposure to mono-GO and multi-GO. The bacterial endotoxin LPS which is known to induce the release of inflammatory factors in DC2.4 cells were used as positive control. As shown in **Figure 3**, LPS remarkably induced the release of the pro-inflammatory cytokines IL-6 and TNF-α in DC2.4 cells. However, Mono-GO and multi-GO hardly induced the release of the IL-6 (**Figures 3A–D**), while obviously increased the production of TNF-α from DC2.4 cells with increasing concentration (**Figures 3E, H**).

**Figure 4:**
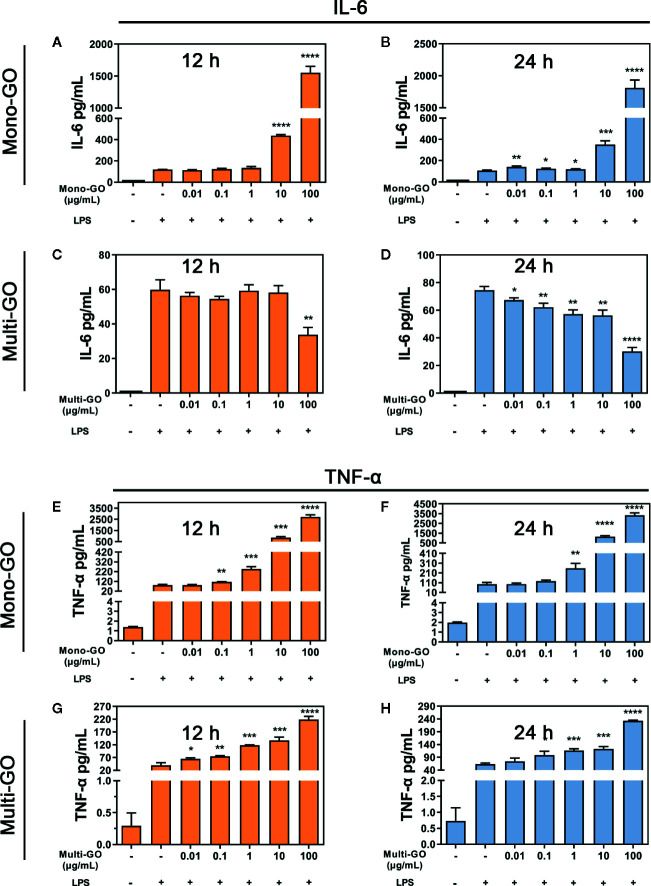
Mono–graphene oxide (GO) or multi-GO promotes or inhibits lipopolysaccharide (LPS)-induced IL-6 and TNF-α cytokine secretion. **(A, B, E, F)** DC2.4 were treated with mono-GO (10 μg/ml, 4 h) before the addition of LPS (1 μg/ml, 12 h or 24 h; n = 3). **(C, D, G, H)** DC2.4 were treated with multi-GO (10 μg/ml, 4 h) before the addition of LPS (1 μg/ml, 12 h or 24 h; n = 3). *P < 0.05; **P < 0.01; ***P < 0.001; ****P < 0.0001 vs. LPS.

To analyze the effect of GO on the immune response by DC2.4 cells, we further evaluated stress in DC2.4 cells caused by foreign materials. As the results of **Figures 4A, B** show, after pretreatment with mono-GO, the release of IL-6 in DC2.4 cells towards LPS stimulation was significantly increased. However, after pretreatment with multi-GO, the release of IL-6 in DC2.4 cells treated with LPS was significantly inhibited (**Figures 4C, D**). Simultaneously, after pretreatment with mono-GO or multi-GO, the release of TNF-α from DC2.4 cells in response to LPS was significantly increased (**Figures 4E–H**). These results indicated that GO was immunotoxic to DC2.4 cells *in vitro* and was able to cause DCs dysfunction.

### RNA-Seq Analysis

**Figure 5:**
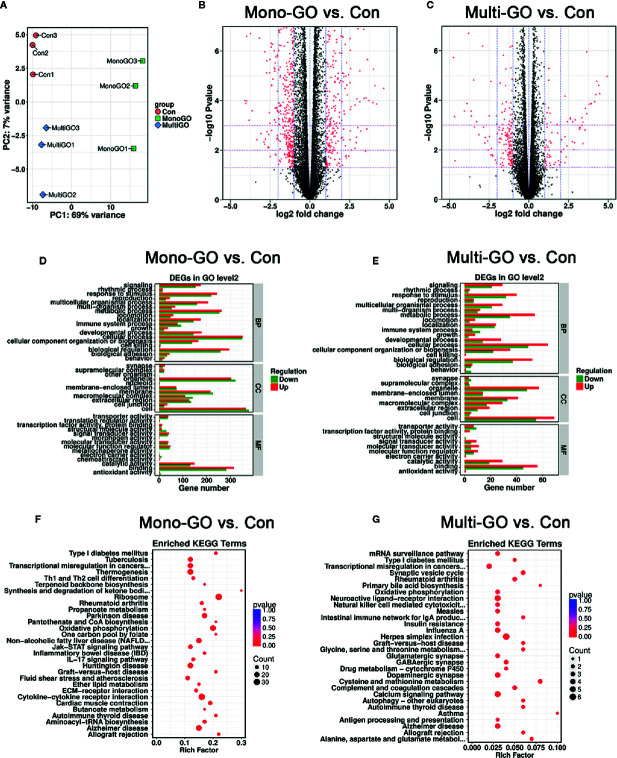
**(A)** Principal component analysis (PCA) plots including all data, three groups [control, mono–graphene oxide (GO), multi-GO] and their biological replicates; control is in pink, mono-GO is in green and multi-GO is in blue. **(B, C)** The volcano map shows that all differentially expressed genes compared to the control group after treatment of DC2.4 cells with mono-GO and multi-GO. (P< 0.05, log_2_ fold change > 1 or log_2_ fold change < -1). **(D, E)** DC2.4 cells were analyzed for Gene Ontology (GO) enrichment at level 2 after mono-GO and multi-GO treatments. **(F, G)** DC2.4 cells were analyzed for KEGG pathway after mono-GO and multi-GO treatments.

We also conducted experiments at the transcriptome level and found that many genes in DC2.4 cells were up-regulated and down-regulated after mono-GO and multi-GO treatment. Through analysis, we found that the top 20 genes that significantly changed after mono-GO and multi-GO treatment of DC2.4 cells were Hsp90ab1, Rplp0, Hspa8, Hdgf, Actb, Ldha, Eef1a1, Rpl4, Fth1, Ftl1, Pkm, P2rx7, Eef2, Pabpc1, Gpnmb, Spp1, LOC108169013, Ncl, Eif4g2, and Lcp1. A Principal Component Analysis (PCA) on transcripts was used to assess the quality of the RNA-seq data. In the PCA plot (**Figure 5A**), three treatments cluster separately from the others. As shown in **Figure 5B**, the volcano map revealed significant changes in the expression level of 1239 genes between mono-GO group and control group (519 up-regulated genes, 720 down-regulated genes, P< 0.05, log_2_ fold change > 1 or log_2_ fold change < -1). As shown in **Figure 5C**, the volcano map also revealed significant changes in the expression level of 261 genes between multi-GO group and control group (91 up-regulated genes, 170 down-regulated genes, P< 0.05, log_2_ fold change < -1 or > 1). Functional annotations against the mouse database revealed series of altered transcriptions involved in BP, CC and MF (**Figures 5D, E**). These results suggested that mono-GO and multi-GO treatment would lead to obvious changes in gene expression profile. In addition, we conducted a KEGG pathway analysis, and the results are displayed in the form of a bubble chart (**Figures 5F, G**). It was shown that mono-GO changed many pathways, such as Th1 and Th2 cell differentiation, IL-17 signaling pathway, cytokine-cytokine receptor interaction, ribosome, oxidative phosphorylation and inflammatory bowel disease (IBD), etc. However, the signal pathways changed by multi-GO were different from mono-GO, such as cysteine and methionine metabolism, asthma, synaptics vesicle cycle, and intestinal immune network for IgA production, etc.

**Figure 6:**
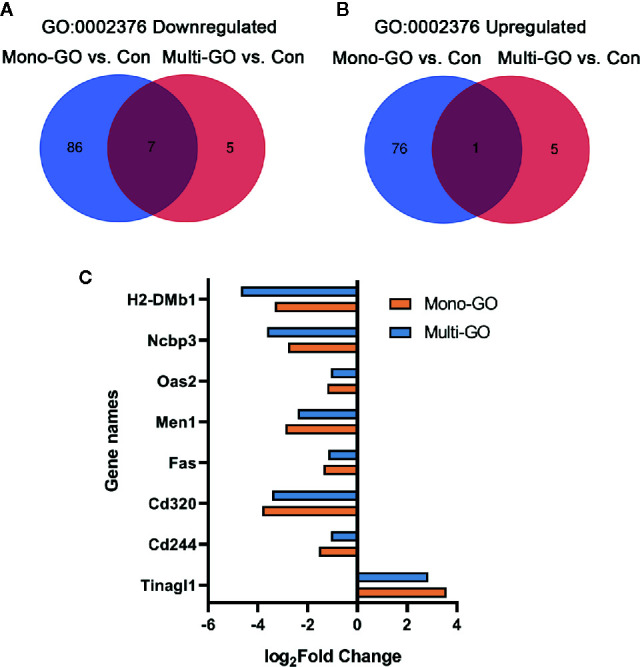
Venn diagram of upregulated and downregulated immune system process (GO: 0002376) after exposure to mono–graphene oxide (GO) and multi-GO compared with control. **(A)** Venn diagram of downregulated immune system process (GO: 0002376). **(B)** Venn diagram of upregulated immune system process (GO: 0002376) **(C)** The fold change in differentially expressed genes shared between mono-GO vs. control and multi-GO vs. control.

In order to analyze the similarities and differences of the effects of mono-GO and multi-GO on immune system process (GO: 0002376), the Venn diagram shown in **Figures 6A, B** plots the overlapping of differentially expressed genes from immune system processes between different treatment groups. Compared with multi-GO treatment group, the mono-GO treatment group had greater influence on the immune response process of DC2.4 cells. We identified 7 shared downregulated genes and 1 shared upregulated gene between mono-GO vs. control and multi-GO vs. control. The shared differentially expressed genes included H2-DMb1, Ncbp3, Oas2, Men1, Fas, Cd320, Cd244, and Tinagl1 which play great important role in the immune system process (**Figure 6C**).

## Discussion

In recent years, because of the unique electronic and physicochemical properties of GFNs, they are widely used in various fields, including nanoelectronics and nanobiomedicine (Thangamuthu et al., 2019). However, safety risk is still a great concern for their application and the potential toxicity of human health need to be addressed. Especially, the interaction of GFNs with the immune system remains undefined. DCs are important components of the immune system and play a key role in regulating the body’s immune function and maintaining the stability of the body’s function. Both the hyperactivity and the underdevelopment of DC’s immune function will lead to the imbalance of the body’s immune function, leading to the occurrence of diseases. In this work, we aimed to determine the effects of mono-GO and multi-GO on DCs using *in vitro* cell model.

According to bioapplication and various toxicity studies of GO (Shao et al., 2017; Hoyle et al., 2018; Pelin et al., 2018; Gurunathan et al., 2019a), we finally selected concentrations of 0.01 μg/ml, 0.1 μg/ml, 1 μg/ml, 10 μg/ml, and 100 μg/ml for *in vitro* toxicity tests. Here, our results showed mono-GO is less toxic to DCs than multi-GO. The cytotoxicity of multi-GO on DC2.4 is concentration dependent after treatment with 24 h or 48 h. Even at low concentrations (0.1 μg/ml), a high cell proliferation inhibition occurred. Differently, the cell viability of DC2.4 showed no obvious difference after mono-GO treatment for 24 h, but appeared to increase after treatment for 48 h at the concentration of 100 μg/ml. These results indicated that the cytotoxicity of GO on DCs was closely related to the layers and incubation time. Previous reports have proved that many factors are affecting the toxicity of graphene in biological system such as concentration, lateral dimension, layers, surface structure and functional groups (Ou et al., 2016). For example, Peruzynska et al. observed the viability of MCF7 cells exposed to single and four layers GO nanoflakes for 48 h. They found that GO nanoflakes with concentrations lower than 50 μg/ml exhibited no obvious cytotoxicity, but at a concentration of 100 μg/ml GO the number of living cells significantly reduced, survival rate of 55.5% for 1-layer graphene oxide, and 52.9% for 4-layer graphene oxide, respectively (Peruzynska et al., 2017). Jia et al. studied the toxicity of three different sizes (small, medium and large) of graphene (G) and graphene oxide (GO) *in vivo* and *in vitro* and they concluded that the toxicity of G and GO is closely related not only to their inherent chemical properties (oxidation state and side dimensions), but also to the exposure concentration, time, and type of toxicity assessment model used (Jia et al., 2019). Gies et al. systematically evaluated the effects of cell type, exposure time, and sheet size of GO on cytotoxicity, and found that the cytotoxicity of GO is closely related to the cell type (Gies and Zou, 2018). Their data showed that GO had the least toxicity to adherent cells, but the most toxicity to suspension cells. They also investigated the effects of three sizes of GO on the cytotoxicity of different cell types. They found that the cytotoxicity was dependent on GO sheet size for NIH 3T3, U87, and A549 cells, while little effect of sheet size on toxicity for RAW 264.7, NB4, and HL60 cell lines.

Previous research reported that the production of ROS is one of the important mechanisms for the toxicity of nanoparticles (Khatri et al., 2013; Seabra et al., 2014; Tang et al., 2018). Nanoparticles interfere with oxidative balance of cell and cause oxidative stress to generate reactive oxygen species, such as superoxide, hydroxyl radical, peroxide radical, hydrogen peroxide, and singlet oxygen (Yan et al., 2017; Lu et al., 2019). A large amount of ROS can destroy cell structure and cell function, and even cause cell death. Xiong et al. studied the mechanism of the toxicity of GO on zebrafish, and they found that GO-induced liver injury was mainly mediated by ROS and MAPK signals (Xiong et al., 2020). Pelin et al. found that few-layer-graphene and GO mediated a significant increase in intracellular ROS production primarily through activation of flavin-based oxidase in human HaCaT skin keratinocytes, and caused significant mitochondrial membrane depolarization and mitochondrial damage (Pelin et al., 2018). In our study, the results showed that 10 μg/ml mono-GO and multi-GO caused the production of ROS in DC2.4 cells, and it was a time-dependent increase within 2 h. Interestingly, 10 μg/ml mono-GO increased intracellular ROS production but did not affect cytotoxicity. The results indicated that ROS generated in a short period of time may not cause cell death. This is similar to previous reports. Chang et al. reported that at lowest concentration of GO was found to induce significant increase in ROS levels in A549 cells without affecting cell viability (Chang et al., 2011). Although the generation of ROS is one of the important mechanisms for cell death caused by nanomaterials, there are still many other cell death-related mechanisms which are responsible for the cytotoxicity induced by nanoparticles such as apoptosis, necrosis, autophagy and necrosis (Ou et al., 2016). In our study, we also found that mono-GO-induced ROS production was more than multi-GO after 30- and 120-min exposure. The reason may be caused by different generation and elimination rates of ROS. Antioxidant enzymes, such as superoxide dismutase or glutathione peroxidase, can reduce and eliminate ROS. The specific mechanism needs further study.

Although mono-GO did not cause cell death, we found an interesting phenomenon in cell morphology. After exposing DC2.4 cells to 10 μg/ml mono-GO for 24 h, the cell morphology changed, cells began to aggregate and grow, and lost contact inhibition. Staining with ghost pen cyclopeptide revealed that compared with the control group, the cells became larger and the cytoplasm became larger and fuller. Hu et al. reported that compared with untreated DCs, DC2.4 cells treated with LPS showed a more mature shape, more extensive dendritic formation and wrinkling (Hu et al., 2012). Perhaps mono-GO promoted DC2.4 activation into mature DCs. The maturation of DCs will lead to the production and release of cytokines, which will trigger immune defense. Previous research suggested that after human DCs are treated with PLGA nanoparticles (NPs) for 24 h, they produce pro-inflammatory cytokines such as IL-6, IL-8, IL-β, and TNF-α. (Barillet et al., 2019). Conversely, in murine BMDCs, NPs had no effect on cytokines secretion. But when cells were treated with lower concentrations of PLGA NPs, it produced IL-6 and TNF-α In our results, there were no effects on IL-6 cytokines secretion, but induced TNF-α cytokine secretion after DC2.4 cells incubated with mono-GO and multi-GO for 24 h and 48 h. And mono-GO induced more secretion of TNF-α cytokines in DC2.4 than multi-GO. TNF-α plays an important role in promoting DCs maturation and immune inflammatory response. Therefore, we speculated that compared with multi-GO, mono-GO could cause a more severe inflammatory response, and then promoted DC2.4 cells to mature. Although study showed that mono-GO and multi-GO had different effects on DC2.4 cytotoxicity and cytokine production, further parameters such as functional characteristics need to be further evaluated.

Effective antigen presentation and T cell activation are highly correlated with cytokine release. Previous research showed that LPS binding to TLR4 activates the downstream NFκB signaling pathway, which stimulates DCs to produce TNF-α and IL-6 inflammatory factors and initiates an adaptive immune response (Rhule et al., 2008; O’Neill et al., 2013). So, we investigated whether GO affected the normal cytokines secretory of DC2.4 in response to LPS. Our results clearly suggested that GO disrupted the release of cytokines TNF-α and IL-6 from DC2.4 cells in response to LPS. We found that mono-GO and multi-GO promoted the release of TNF-α cytokines in LPS-induced DC2.4 cells. In contrast, mono-GO LPS-induced IL-6 release in DC2.4 cells, but multi-GO inhibits LPS-induced IL-6 release. As previously studied, exposing DCs to GO to achieve responses to TNF-α and IL-6 may control TH1 response (Barillet et al., 2019), which is consistent with the results of TH1 response induced by delivery of model antigens through PLGA particles (Newman et al., 1998). The decreased level of IL-6 in multi-GO treated cells after LPS stimulation was likely due to the cell viability inhibition caused multi-GO. Overall, our results suggested that GOs changed the inflammatory response of DCs to LPS stimulation. Further investigation is needed to show whether the release of cytokines by GO-induced DCs changes their ability to initiate T-cell dependent immune responses.

In order to further study the immunotoxicity of mono-GO and multi-GO, we performed whole transcriptome sequencing analysis. Recently, high-throughput screening methods have been widely used to assess the toxicity of nanoparticles. Deng et al. employed whole-transcriptome profiling to identify 2116 differentially expressed genes between the zebrafish larvae exposed graphene quantum dots (GQDs) groups and the control group and found that GQDs significantly up-regulated most genes in the acute inflammatory response and detoxification process (Deng et al., 2018). In order to better understand the effects of cadmium telluride (CdTe) QD on central nervous system toxicity, Wu et al. performed a high-throughput sequencing to analyze the changes in the rat hippocampal genome caused by two sizes of CdTe QDs and found that compared to 2.2 nm CdTe QDs, 3.5 nm CdTe QDs caused severe inflammatory and immune responses in the hippocampus of rats (Wu T. S. et al., 2018). Our bioinformatics data showed that both mono-GO and multi-GO caused changes in the transcriptome level of DC2.4 cells, and found 1,239 differentially expressed genes (DEGs) were between the mono-GO and control and 261 DEGs were between the multi-GO and control. The result suggested that mono-GO exposure might have more severe effects on DC2.4 cells than multi-GO at the transcriptome level. The effect of GO on the transcriptome level of DCs may be related to factors such as the number of layers and lateral size of GO. We also found that 7 shared downregulated genes (H2-DMb1, Ncbp3, Oas2, Men1, Fas, Cd320, and Cd244) and 1 shared upregulated genes (Tinagl1) between mono-GO vs. control and multi-GO vs. control on immune system process (GO: 0002376). All of these genes play great important role in the immune system process. For example, previous study found that activated Fas signaling in DCs induced cytokine secretion and played an important role in adjusting T cell activation, proliferation, differentiation and inflammation response (Han M. M. et al., 2018; Zu Horste et al., 2018). Based on these results, we inferred that at the transcriptome level, mono-GO produced a stronger immune response than multi-GO on DCs, although they share similar changes for some genes. Gurunathan et al. assessed the immunotoxicity of GO and vanillin-functionalized GO (V-rGO) on THP-1 cells, a human acute monocytic leukemia cell line and they concluded that V-rGO showed significant effects on immunotoxicity compared to GO because of the sharp edges, chemical composition, charge transfer, different carbon to oxygen ratio, and functional groups present on V-rGO (Gurunathan et al., 2019b). In summary, the immunotoxic response of graphene oxide to cells in this study is closely related to the layers, size, and degree of oxidation of graphene oxide.

In our study, mono-GO and multi-GO both had different degrees of toxicity to DC2.4 cells. Previously, it has been shown that PEGylated nanomaterials are biocompatible and do not cause a severe immune response compared to the original materials. For example, Cicuéndez et al. studied the effect of GBNs on macrophages *in vitro*, and found that the inflammatory response is related to particle size and surface modification (Cicuendez et al., 2020). Smaller-sized nanomaterials induced the pro-inflammatory response of macrophages, while PEGlayted GO did not activate this response. However, the research by Luo et al. contradict these results (Luo et al., 2017). They found that PEGylated GO would not be internalized into cells, but would cause the release of stronger cytokines in peritoneal macrophages compared to GO. Next, they studied the mechanism of GO interaction with cells through atomistic molecular dynamics simulations, and they found GO preferentially adsorb onto and/or partially insert into cell membranes and activated downstream signaling pathways. Further experiments showed that PEGylated GO stimulated the secretion of cytokines by enhancing the integrin β8-related signaling pathway. Wu et al. prepared five kinds of PEGlayted GO with differing surface charge and degrees of oxidation and compared their toxicity to ocular surface cells and intraocular cells *in vitro* (Wu W. et al., 2018). It was found that cytotoxicity was related to the oxidation level of nanomaterials, but the surface charge of nanomaterials had no significant effect. Among them, high oxidative levels of PEGlayted GO resulted in higher cytotoxicity. In summary, PEGlayted GO may reduce the toxicity of cells, but we need to conduct subsequent evaluations of the size, number of layers, and surface charge of PEGlayted GO for better biological applications.

## Conclusion

In summary, here we investigated the immunotoxicity of mono-GO and multi-GO on DC2.4 cells. We found that both mono-GO and multi-GO significantly induced ROS production in DC2.4 cells, but mono-GO had less effect on DC2.4 cell viability compared to multi-GO. Interestingly, mono-GO enabled DC2.4 cells to aggregate and change the cell morphology, while multi-GO had no similar effect. In addition, mono-GO and multi-GO hardly induced the release of the IL-6, while obviously increased the production of TNF-α from DC2.4 cells. Mono-GO and multi-GO promoted TNF-α production in DC2.4 cells induced by LPS. In contrast, mono-GO LPS-induced IL-6 release in DC2.4 cells, but multi-GO inhibits LPS-induced IL-6 release. Gene expression profiling showed that both mono-GO and multi-GO caused changes in the transcriptome level of DC2.4 cells, and mono-GO caused more altered genes than multi-GO. There are some similarities and differences of the effects of mono-GO and multi-GO on immune system process (GO: 0002376). These results indicated that GO had immunotoxicity to DC2.4 cells and was able to cause DCs dysfunction. These results suggest their potential immunotoxicity effects on DCs should be taken into full consideration in their biological applications although GO has many advantages. This is of great significance for the subsequent biological and clinical applications of GO.

## Data Availability Statement

The Illumina reads generated in this study are available at the NCBI Sequence Read Archive browser (http://ncbi.nlm.nih.gov/sra, accession no. SRR10967877, SRR10967878, SRR10967879, SRR10967880, SRR10967881, SRR10967882, SRR10967883, SRR10967884 and SRR10967885). We declare that the Raw sequencing data is available through the NCBI Sequence Read Archive under Project ID PRJNA602127 (http://trace.ncbi.nlm.nih.gov/Traces/sra/).

## Author Contributions 

GL conceived and designed the experiments. ZY, YP, WZ, DX, and DL experimented and collected the data. ZY, TC, and LL performed a statistical analysis of all the data. ZY, TC, and LL analyzed the data and drafted the manuscript. GL and XW made a strict modification of the manuscript. All authors contributed to the article and approved the submitted version.

## Funding

The project is supported by the grant from National Natural Science Foundation of China (NSFC) (No. 21677102), the grant from Shenzhen Basic Research Project (No. JCYJ20190808153803952), the grant from Discipline Layout project of Shenzhen Science and Technology innovation committee (JCYJ20170818092553608), and the grant from Guangdong Basic and Applied Basic Research Foundation (NO. 2019A1515110342).

## Conflict of Interest

The authors declare that the research was conducted in the absence of any commercial or financial relationships that could be construed as a potential conflict of interest.
